# Outcomes and Prognostic Factors of Pulmonary Hypertension Patients Undergoing Emergent Endotracheal Intubation

**DOI:** 10.1177/08850666221118839

**Published:** 2022-08-08

**Authors:** Andrew W. Hong, William Toppen, Joyce Lee, Holly Wilhalme, Rajan Saggar, Igor Z. Barjaktarevic

**Affiliations:** 1 David Geffen School of Medicine at UCLA, Los Angeles, CA, USA; 2 Department of Medicine, 8783 University of California, Los Angeles, CA, USA; 3 Division of General Internal Medicine and Health Services Research, 12222 David Geffen School of Medicine, Los Angeles, CA, USA; 4 Department of Pulmonary and Critical Care, UCLA Medical Center, Los Angeles, CA, USA

**Keywords:** endotracheal intubation, pulmonary hypertension, right heart failure, critical care, mechanical ventilation, vasopressor agents, acute kidney injury

## Abstract

**Background:** Emergent endotracheal intubations (ETI) in pulmonary hypertension (PH) patients are associated with increased mortality. Post-intubation interventions that could increase survivability in this population have not been explored. We evaluate early clinical characteristics and complications following emergent endotracheal intubation and seek predictors of adverse outcomes during this post-intubation period. **Methods:** Retrospective cohort analysis of adult patients with groups 1 and 3 PH who underwent emergent intubation between 2005-2021 in medical and liver transplant ICUs at a tertiary medical center. PH patients were compared to non-PH patients, matched by Charlson Comorbidity Index. Primary outcomes were 24-h post-intubation and inpatient mortalities. Various 24-h post-intubation secondary outcomes were compared between PH and control cohorts. **Results:** We identified 48 PH and 110 non-PH patients. Pulmonary hypertension was not associated with increased 24-h mortality (OR 1.32, 95%CI 0.35-4.94, *P* = .18), but was associated with inpatient mortality (OR 4.03, 95%CI 1.29-12.5, *P* = .016) after intubation. Within 24 h post-intubation, PH patients experienced more frequent acute kidney injury (43.5% vs. 19.8%, *P* = .006) and required higher norepinephrine dosing equivalents (6.90 [0.13-10.6] mcg/kg/min, vs. 0.20 [0.10-2.03] mcg/kg/min, *P* = .037). Additionally, the median P/F ratio (PaO_2_/FiO_2_) was lower in PH patients (96.3 [58.9-201] vs. 233 [146-346] in non-PH, *P* = .001). Finally, a post-intubation increase in PaCO_2_ was associated with mortality in the PH cohort (post-intubation change in PaCO_2_ +5.14 ± 16.1 in non-survivors vs. −18.7 ± 28.0 in survivors, *P* = .007). **Conclusions:** Pulmonary hypertension was associated with worse outcomes after emergent endotracheal intubation than similar patients without PH. More importantly, our data suggest that the first 24 hours following intubation in the PH group represent a particularly vulnerable period that may determine long-term outcomes. Early post-intubation interventions may be key to improving survival in this population.

## Introduction

Managing pulmonary hypertension (PH) patients in a critical care setting is challenging as routine interventions such as volume resuscitation and mechanical ventilation are often complicated by severe hemodynamic instability and right ventricular (RV) failure, which is the leading cause of death.^1–3^ Thus, experts recommend avoiding endotracheal intubation (ETI) unless necessary.^4^ Brief periods of hypoxemia and hypercapnia may provoke RV failure by increasing pulmonary vascular resistance (PVR), contributing to the pronounced effects of induction medications on hemodynamic instability.^5–7^ For these reasons, there is an increased risk of mortality in PH patients who undergo ETI and, in particular, emergent intubations.^8–10^ While alternate approaches such as awake intubation have been suggested,^10^ we have yet to study the efficacy of specific interventions in the immediate post-intubation period that could increase survivability. To our knowledge, there are no cohort studies comparing outcomes in ETI between PH patients and non-PH patients to date.

In this retrospective cohort study, we analyze clinical outcomes of PH patients requiring intubation and then subsequent mechanical ventilation and compare them to the outcomes of matched non-PH patients. We aim to further evaluate the clinical characteristics and complications following emergent endotracheal intubation and identify predictors of adverse outcomes during this immediate, post-intubation period. We hypothesize that the presence of PH would independently increase the risk of short and long-term mortality, and PH patients will have more adverse events in the immediate post-intubation period than non-PH patients. With this data, we hope to lay the groundwork to identify specific strategies that could increase the survival of PH patients undergoing emergent ETI in the intensive care unit (ICU) setting.

## Materials and Methods

### Identification of PH Patients and a Matched Non-PH (Control) Cohort

We proposed a retrospective cohort study of PH patients and matched non-PH patients who underwent emergent ETIs from 2005 through 2021 at a large tertiary academic medical center. Study participants with PH were selected using the classic definition of mean pulmonary arterial pressure (mPAP) ≥25 mmHg at rest.^4^ During our database search, inclusion criteria consisted of either group 1 or 3 PH patients (pulmonary arterial hypertension and PH secondary to lung disease, respectively) who required emergent intubation. We searched for group 1 or 3 patients to focus on pre-capillary PH subtypes. Additionally, our institution is a major referral center for pulmonary fibrosis, leading to an increased representation of group 3 PH subjects.

**Figure 1.**
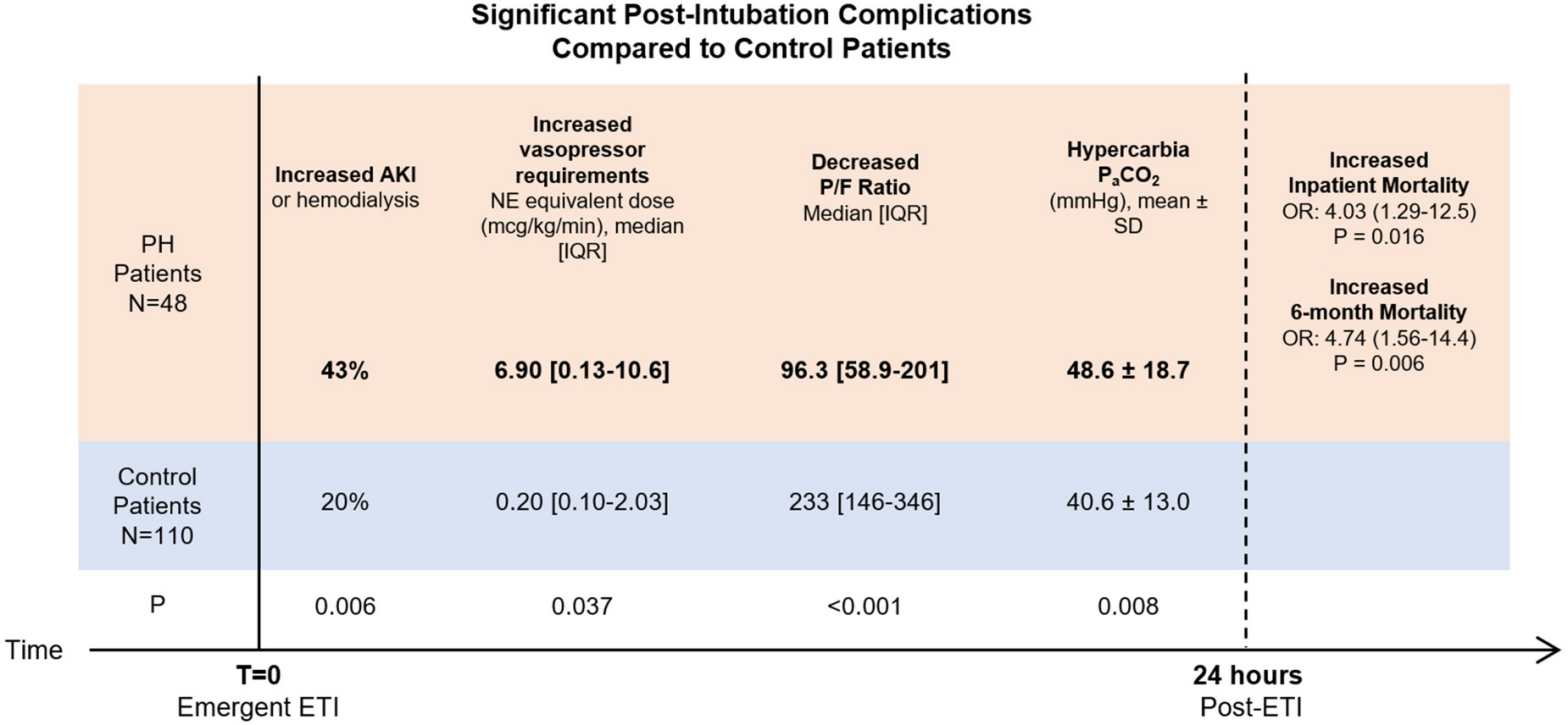
Summary illustration of early, post-intubation complications 24-h after intubation in pulmonary hypertension patients compared to control patients.

For the control cohort, we queried our database for non-PH patients who also underwent emergent ETI matched by Charlson comorbidity index (CCI) with a goal cohort ratio of 1:3 controls. While determining the method of identifying matched control individuals was challenging, we ultimately decided to match our reference group of patients with CCI as it is a well-validated prognostic predictor of overall mortality.^11^ As both our study and reference groups were ill and required emergent intubations, we aimed to identify potential adverse outcomes that would be more attributable to pulmonary hypertension independent of other significant chronic, comorbid diseases (Figure 1).

Summary depiction of our significant findings. Out of the various secondary outcome metrics measured in Table 2, the following complications in pulmonary hypertension patients were significantly different compared to the non-pulmonary hypertension cohort.

We included all urgent and emergent cases, including patients intubated due to cardiac arrest. Lastly, we excluded patients who were intubated and then placed on extracorporeal membrane oxygenation (ECMO). Our study was reviewed and approved by our institutional review board, Office of the Human Research Protection Program (OHRPP), IRB#15-001844.

### Method of ETI

All intubations were done via performing physician preference, though our standard protocol was rapid sequence intubation (RSI) in emergent settings. The general protocol for RSI included pre-oxygenation of patients with high FiO_2_ and ventilatory assistance with a bag valve mask (BVM). The choice of induction medications was at the discretion of the intubating physician. Endotracheal tube placement was assisted with either direct laryngoscopy or video laryngoscope.

### Outcomes

The primary outcomes were short-term mortality defined in 3-h and 24-h time intervals post-intubation and inpatient mortality. Long-term 28-day and 6-month mortalities were also assessed. Secondary outcomes included various outcome metrics within the 24-h post-intubation period. Maximum vasopressor dosing within 24-h post-intubation was expressed as the total sum of maximum norepinephrine (NE) equivalents using conversion factors derived from a recent study conducted by Khanna *et al.*^12^

### Sub-Group Analyses

Patients who had immediate pre- and post-intubation arterial blood gases (ABG) were included in subgroup analyses. We assessed differences in PaCO_2_, PaO_2_, and pH between pulmonary hypertension and control cohorts, further subcategorized into patients who died or lived during their hospital stay (inpatient mortality). Pre and post-intubation ABG metrics were calculated as follows: Post-intubation value – pre-intubation value.

### Statistical Analyses

Binary outcomes between PH and control cohorts were analyzed with Fisher's exact chi-square, while continuous variables were analyzed with t-tests. Odds of mortality were assessed with univariate logistic regressions, utilizing propensity score weighting to account for significantly different baseline characteristics between cohorts. These propensity score-weighted logistic regressions were specifically used to evaluate the association between pulmonary hypertension and mortality after emergent ETI. *P* values <.05 were considered statistically significant.

## Results

Initially, 113 control patients were identified; 2 patients were excluded because they were intubated for ECMO, and 1 patient was excluded due to non-emergent intubation. The final study cohort consisted of 48 PH patients with 110 control patients.

The combined cohort mean age was 55.2 ± 13.2, 53% were female, and 53% were Caucasian. For both cohorts, the mean CCI was 6.0 ± 3.0. The significant differences between groups were BMI, Glasgow coma score (GCS), heart rate (HR), pre-intubation vital signs: respiratory rate (RR), mean arterial pressure (MAP) <65, and sodium levels pre-intubation. Complete patient characteristics are tabulated in Table 1. The following characteristics were included for propensity score weighting: age, CCI, gender, race, BMI, GCS, pre-intubation vitals (HR, RR, MAP, temperature), and pre-intubation labs – sodium, potassium, creatinine, hematocrit (Hct). Table 2 demonstrates improved balance in baseline patient characteristics between PH and control patients after propensity score weighting.

**Table 1.** Baseline Patient Characteristics.

Characteristic	PH (N = 48)	Control (N = 110)	*P*
Reason for Intubation, (%)			
Airway Protection	4 (8%)	36 (33%)	
Cardiac Arrest	8 (17%)	10 (9%)	
Acute respiratory failure	1 (2%)	7 (6%)	
Aspiration	0 (0%)	3 (3%)	
Hypercapnia	3 (6%)	4 (4%)	
Hypotension	0 (0%)	1 (1%)	
Hypoxemia/hypoxia	21 (44%)	18 (17%)	
Post-ROSC (w/ pulses)	0 (0%)	2 (2%)	
Respiratory distress	11 (23%)	28 (26%)	
Age at Intubation, (mean ± SD), years	54 ± 15	56 ± 13	.37
Female, (%)	26 (54%)	58 (54%)	1.00
White or Caucasian, (%)	29 (60%)	56 (51%)	.30
BMI, (mean ± SD), kg/m^2^	26 ± 8	28 ± 6	.05
Charlson Score, (mean ± SD)	6 ± 3	6 ± 3	.24
Pulmonary Hypertension Characteristics			
WHO Class, (%)			
1	31 (65%)		
3	17 (35%)		
Most recent PASP (mean ± SD), mm Hg	78 ± 24		
Echocardiogram Data			
Most recent Tricuspid Regurgitant Jet Velocity (TRV) (mean ± SD), m/s	3.85 ± 0.84		
Right Ventricular Dysfunction, (%)	33 (70%)		
Severity, if commented on report			
Mild	10 (33%)		
Moderate	8 (27%)		
Severe	12 (40%)		
Right Heart Chamber Enlargement, (%)	37 (79%)		
Severity, if commented on report			
Mild	5 (20%)		
Moderate	8 (32%)		
Severe	12 (48%)		
On PH Medication prior to admission, (%)	30 (64%)		
Pre-intubation vital signs, (%)			
HR >100 or <50	31 (65%)	46 (44%)	.024
RR >22	39 (81%)	48 (46%)	<.001
Temp >38 or <36	13 (27%)	20 (21%)	.41
MAP <65	17 (35%)	20 (19%)	.041
GCS, (median [IQR])	15 [10-15]	12 [6-15]	.001
Pre-intubation Labs			
Na Prior, (mean ± SD), mmol/L	136 ± 6	139 ± 7	.004
K Prior, (mean ± SD), mmol/L	4.3 ± 0.7	4.1 ± 0.9	.15
Cr Prior, (median [IQR]), mg/dL	1.2 [0.9-1.7]	1.0 [0.6-1.7]	.17
Hct Prior, (mean ± SD), %	11.1 ± 2.9	10.6 ± 2.6	.36

**Table 2.** Baseline Patient Characteristics After Propensity Score Weighting.

		Unweighted			Weighted	
Characteristic	PH (N = 48)	Control (N = 110)	*P*	PH (N = 48)	Control (N = 110)	*P*
Age at Intubation, mean	54	56	.37	53.4	54.1	.83
Female, (%)	26 (54%)	58 (54%)	1.00	26 (54%)	61 (56%)	.83
White or Caucasian, (%)	29 (60%)	56 (51%)	.30	29 (60%)	50 (45%)	.14
BMI, (mean), kg/m^2^	26	28	.05	25.9	26.7	.60
Charlson Score, mean	6.0	6.0	.24	5.7	6.5	.17
Pre-intubation Vital Signs						
HR >100 or <50, (%)	31 (65%)	46 (44%)	.024	30 (62%)	84 (76%)	.17
RR >22, (%)	39 (81%)	48 (46%)	<.001	38 (80%)	95 (86%)	.40
Temp >38 or <36, (%)	13 (27%)	20 (21%)	.41	12 (26%)	17 (15%)	.20
MAP <65, (%)	17 (35%)	20 (19%)	.041	16 (34%)	33 (30%)	.66
GCS, (mean)	15	12	.001	12.6	13.7	.09
Pre-intubation Labs						
Na Prior, (mean), mmol/L	136	139	.004	136	135	.50
K Prior, (mean), mmol/L	4.3	4.1	.15	4.33	4.19	.33
Log Cr Prior, (mean)	0.12	0.05	.24	0.12	0.22	.15
Hct Prior, (mean), %	11.1	10.6	.36	35.8	31.8	.01

Unweighted baseline patient characteristics are listed on the left, which are identical to Table 1. New propensity score weighted values on the right.

Table 3 displays the frequency of comorbidities that constitute the Charlson Comorbidity Index within the PH and control cohorts. Of note, there was a greater number of individuals with a history of myocardial infarction in the control cohort (28.2% vs. 10.5% in the PH cohort, *P* = .014). Though more PH patients had congestive heart failure (50%), this was not significantly different from the control patients (37.3%, *P* = .13). Otherwise, control patients had greater incidences of comorbidities such as peripheral vascular disease, history of cerebrovascular accident or transient ischemic attacks, connective tissue disease, liver disease, hemiplegia, and solid tumors.

**Table 3.** Breakdown of Charlson Score Comorbidities Between Pulmonary Hypertension and Control Patients.

Comorbidity	PH (N = 48)	Control (N = 110)	*P*
History of Myocardial Infarction, (%)	5 (10.4%)	31 (28.2%)	.014
Congestive Heart Failure, (%)	24 (50.0%)	41 (37.3%)	.13
Peripheral Vascular Disease, (%)	1 (2.1%)	33 (30.0%)	<.001
History of cerebrovascular accident or transient ischemic attacks, (%)	3 (6.3%)	42 (38.2%)	<.001
Dementia, (%)	1 (2.1%)	9 (8.2%)	.15
Chronic Pulmonary Disease, (%)	48 (100%)	49 (44%)	<.001
Connective Tissue Disease, (%)	14 (29%)	8 (7.3%)	<.001
Peptic Ulcer Disease, (%)	3 (6.3%)	10 (9.1%)	.55
Liver Disease, (%)			<.001
Mild (no portal HTN)	1 (2.1%)	47 (43%)	
Moderate or Severe	9 (18%)	0 (0%)	
None	38 (79%)	63 (57%)	
Diabetes Mellitus, (%)			.007
End-organ Damage	2 (4.2%)	0 (0%)	
None or Diet-Controlled	37 (77%)	69 (62%)	
Uncomplicated	9 (18%)	41 (37%)	
Hemiplegia, (%)	0 (0%)	16 (14%)	.005
Moderate to Severe Chronic Kidney Disease, (%)	11 (23%)	40 (36%)	.096
Solid Tumor, (%)			.002
None	45 (94%)	77 (70%)	
Localized	3 (6.3%)	21 (19%)	
Metastatic	0 (0%)	12 (11%)	
Leukemia, (%)	0 (0%)	3 (2.7%)	.25
Lymphoma, (%)	1 (2.1%)	0 (0%)	.13
AIDS, (%)	0 (0%)	2 (1.8%)	.35

There is one noteworthy distinction that warrants discussion regarding the CCI's definition of chronic pulmonary disease. During the development and validation phase of the CCI, chronic pulmonary disease was defined as the presence of dyspnea with moderate activity, not to be confused with chronic obstructive pulmonary disease (COPD).^13^ For this reason, every PH patient was listed to have chronic pulmonary disease as they qualified under the past definition that was used to validate the CCI.

Indications for intubation are listed in Table 1. Most PH patients were intubated for hypoxemia/hypoxia (44%) and respiratory distress (23%). Most control patients were intubated for airway protection due to altered mental status, seizure, hematemesis/hemoptysis, or trauma (33%). 17% of PH and 9% of control patients were intubated due to cardiac arrest. Per our chart review, none of the intubations were complicated by a lost airway or endotracheal tube misplacement.

### Pulmonary Hypertension and Its Association with Mortality After Intubation

After propensity score weighting, logistic regressions analysis showed no significant association between PH and mortality in the 3-h (OR 0.41, 95%CI 0.07-2.21, *P* = .30) or 24-h (OR 1.32, 95%CI 0.35-4.94, *P* = .18) periods after intubation. PH was also not associated with increased 28-day mortality (OR 2.07, 95%CI 0.55-7.79, *P* = .27). However, PH was significantly associated with inpatient mortality (OR 4.03, 95%CI 1.29-12.5, *P* = .016) and 6-month mortality (OR 4.74, 95%CI 1.56-14.4, *P* = .006) after intubation.

### Short-Term Outcomes 24 h After Endotracheal Intubation

Within 24 h post-intubation, PH patients had a greater incidence of acute kidney injury (AKI) or need for hemodialysis (43.5% vs. 19.8%, *P* = .006) than control patients. PH patients also required higher number of simultaneous vasopressor medications used (1.68 ± 1.30 vs. 1.04 ± 1.01, *P* = .004) and received higher maximum norepinephrine equivalent dosages in the 24-h period post-intubation (median: 6.90 [0.13-10.6] vs. 0.20 [0.10-2.03] mcg/kg/min NE equivalents, *P* = .037). Of the 10 PH individuals who died within 24-h post-intubation, 3 (30%) had new cardiac arrhythmias, while none of these individuals required intubation for cardiac arrhythmia or arrest. Maximum lactate within 24 h post-intubation was similar between both cohorts (median: 19.5 [11.5-55.5] mg/dL vs. 28.0 [16.0-55.0] mg/dL in the PH and control groups, respectively, *P* = .40).

At 24 h post-intubation, mean required FiO_2_ was higher in PH patients (66% ± 22% vs. 49% ± 21% in control, *P* = .001) and mean P/F ratio (PaO_2_/FiO_2_) was lower in PH patients (96.3 [58.9-201] vs. 233 [146-346] in control, *P* = .001). The full table of secondary outcomes in the 24-h post-intubation period is tabulated in Table 4.

**Table 4.** Secondary Outcomes Table: Short-Term Outcomes Within 24 h Post-Intubation.

Outcome	PH (N = 48)	PH “n”	Control (N = 110)	Control “n”	*P*
Hypothermia (<34 °C), (%)	2 (8.7%)	23	9 (8.5%)	106	1.00
GCS, (mean ± SD)	8.83 ± 3.82	18	7.83 ± 3.69	107	.29
PEEP, (mean ± SD), cmH2O	7.25 ± 3.27	20	6.31 ± 2.55	103	.15
P/F Ratio, (median [IQR])	96.3 [58.9-201]	31	233 [146-346]	79	<.001
FiO_2_, (mean ± SD)	66% ± 22%	22	49% ± 22%	103	<.001
Total Number of Simultaneous Vasopressors, (mean ± SD)	1.68 ± 1.30	31	1.04 ± 1.01	105	.004
Max NE Equivalent Dose, (median [IQR]), mcg/kg/min	6.90 [0.13-10.6]	40	0.20 [0.10-2.03]	70	.037
Max NE, (mean ± SD), mcg/kg/min	0.50 ± 0.42	16 (52%)	0.55 ± 1.36	55 (52%)	.88
Max Epinephrine, (mean ± SD), mcg/kg/min	0.29 ± 0.37	6 (19%)	0.56 ± 0.50	11 (10%)	.26
Max Vasopressin, (mean ± SD), units/hr	3.44 ± 0.81	11 (35%)	3.23 ± 0.92	16 (15%)	.55
Max Dopamine, (mean ± SD), mcg/kg/min	10.0 ± 8.89	3 (10%)	10.8 ± 5.90	12 (11%)	.85
Max Phenylephrine (mean ± SD), mcg/min	170 ± 42.4	2 (6%)	108 ± 84.8	14 (13%)	.34
Number of new arrhythmias, (%)	7 (30%)	23	46 (43%)	107	.38
Need for blood/platelet transfusions, (%)	6 (27%)	22	41 (40%)	103	.34
Change in Cr pre- and post-intubation, (mean ± SD)	−0.15 ± 1.66	36	−0.23 ± 1.40	103	.79
AKI, (%)	15 (38%)	39	18 (17%)	106	.013
AKI or HD, (%)	17 (43%)	39	21 (20%)	106	.006
AST, (median [IQR]), IU/L	37.0 [19.0-91.0]	17	55.5 [26.0-244]	76	.17
ALT, (median [IQR]), IU/L	16.0 [9.00-39.0]	15	44.0 [15.5-139]	76	.038
Max lactate (median [IQR]), mg/dL, normal: 5-25 mg/dL	19.5 [11.5-55.5]	16	28.0 [16.0-55.0]	75	.40
Troponin Peak, (median [IQR]), ng/mL, normal: <0.1 ng/mL	0.28 [0.06-1.45]	20	0.17 [0.04-1.80]	50	.82
Change in troponin pre- and post-intubation (mean ± SD), Δng/mL	+0.67 ± 1.16	15	+67.8 ± 325	30	.43

All outcomes were measured in the immediate 24-h post-intubation period. Not all data were available in the electronic health records; the “n” column denotes the total number of data points available for the analysis of each outcome variable. Percentages refer to the number of available data points.

### Arterial Blood Gas Analysis, Ventilation, and Oxygenation After ETI

Pre- and post-intubation ABG values for 35 PH and 87 control patients were available for the following subgroup analysis. Immediately before intubation, the average PaCO_2_ in the PH cohort was 49.9 ± 25.1 mmHg versus 41.1 ± 16.0 mmHg in the control group (*P* = .07). PH patients were more hypercapnic than control patients immediately post-intubation (48.6 ± 18.7 vs. 40.6 ± 13.0, *P* = .008). An increase in PaCO_2_ post-intubation in PH patients was associated with inpatient mortality (+5.14 ± 16.1 in PH who died vs. −18.7 ± 28.0 in PH who lived, *P* = .007). In control patients, increases in post-intubation PaCO_2_ were not associated with inpatient mortality (−4.6 ± 17.2 in those who died vs. −0.39 ± 10.4 in those who lived, *P* = .34). Within both patient populations, pre and post-intubation PaO_2_ changes were not associated with inpatient mortality. Full results are tabulated in Table 5.

**Table 5.** Arterial Blood Gas Subgroup Analyses by Inpatient Mortality.

Subgroup	PH (N = 35)			Control (N = 87)		
Inpatient Mortality	Lived	Died	*P*	Lived	Died	*P*
Pre-intubation ABGs						
P_a_CO_2_, (mean ± SD), mmHg	60.2 ± 37.7	46.1 ± 19.1	.17	43.1 ± 17.8	38.6 ± 13.7	.35
P_a_O_2_, (mean ± SD), mmHg	64.3 ± 9.97	74.6 ± 42.7	.50	126 ± 69.9	88.4 ± 43.4	.04*
pH, (mean ± SD)	7.31 ± 0.16	7.36 ± 0.10	.37	7.42 ± 0.41	7.36 ± 0.10	.49
Post-intubation ABGs						
P_a_CO_2_, (mean ± SD), mmHg	45.2 ± 16.9	50.8 ± 20.0	.42	39.9 ± 12.0	41.5 ± 14.2	.56
P_a_O_2_, (mean ± SD), mmHg	95.6 ± 65.1	106 ± 77.9	.67	181 ± 106	148 ± 116	.18
pH, (mean ± SD)	7.37 ± 0.10	7.31 ± 0.14	.17	7.36 ± 0.10	7.31 ± 0.15	.05
Pre- and post-intubation changes						
ΔP_a_CO_2_, (mean ± SD)_,_ ΔmmHg	−18.7 ± 28.0	+5.14 ± 16.1	.007**	−4.6 ± 17.25	−0.39 ± 10.4	.34
ΔP_a_O_2_, (mean ± SD)_,_ ΔmmHg	+45.2 ± 72.1	+19.0 ± 44.2	.23	+35.0 ± 101	+48.5 ± 73.5	.62
ΔpH, (mean ± SD)	+0.11 ± 0.16	−0.07 ± 0.10	.001**	−0.04 ± 0.42	−0.01 ± 0.13	.73

Patients who had documented arterial blood gas results, both immediately preceding their emergent intubation (pre-intubation) and immediately afterward (post-intubation). All arterial blood gases were collected within 24 h of the intubation event. Pre- and post-intubation changes were calculated as such: post-intubation value – pre-intubation value. Values were further subcategorized by inpatient mortality.

## Discussion

In this retrospective analysis of patients with pulmonary hypertension who underwent emergent ETI compared to a matched control cohort, we observed increased vasopressor requirements, hypoxemia, hypercapnia, and AKI in the early post-intubation period in our cohort of PH patients alongside overall poor survival in PH patients. Despite a lack of increased 3 or 24-h mortality, our results confirm previous data relating pulmonary hypertension as an independent predictor of inpatient morbidity and long-term mortality.^2,14–16^ To our knowledge, this is the first study that analyzes the immediate post-intubation period in PH patients with direct comparison to a matched control cohort. In contrast, most studies characterize the pathophysiology of PH in intubation and mechanical ventilation and propose strategies to prevent RV failure.^1,17^ Our analysis contributes to current knowledge with the identification of the post-intubation period as a vulnerable time for PH patients, and we propose that these early post-intubation complications may contribute to their overall adverse outcomes.

The PH cohort did not have technical endotracheal intubation complications nor increased death rate in the immediate post-intubation period, yet it is plausible that any ETI complications could arise beyond the initial airway securement. With ETI, a spectrum of hemodynamic issues could arise, such as hypotension due to systemic anesthesia and decreased venous return and RV function when transitioning to positive-pressure ventilation or administering fluids.^18–20^ Though our retrospective study cannot determine any causality, we believe that these hemodynamic alterations that occur during intubation may significantly impact longer-term outcomes, especially in pulmonary hypertension patients with an already tenuous hemodynamic status. Thus, we argue that safe intubation practices are crucial, such as adequate pre-oxygenation, judicious use of hypotension-inducing agents, cautious use of intravenous fluids, early vasopressor use to ensure tight maintenance of desired blood pressure, and anticipating decreased venous return when suddenly transitioning to mechanical positive pressure ventilation, which the latter could be alleviated with noninvasive positive pressure ventilation (NIPPV).^21^ Overall, transitioning to mechanical ventilation in critically sick PH patients may require safe, efficient strategies with a focus on minimizing hemodynamic alterations and preventing hypoxemia.^10^

Though definitive intubation approaches that decrease the risk of hemodynamic collapse have yet to be recognized, there may be a few emerging approaches.^6,22^ Our previous study of a small group of patients using awake intubation bridged by noninvasive ventilation demonstrated a promising technique in reducing major complications from intubation such as AKI, despite the need for significant vasopressor support in 5 of 9 patients (55.6%).^10^ Other possible solutions include using ketamine or etomidate for induction which may prevent profound hypotension and significantly decrease the rate of hemodynamic collapse.^23^ Also, awake fiberoptic intubation may circumvent the need for deep anesthetic sedation or be done in conjunction with dexmedetomidine as minimal changes in MAP were observed.^10,24^

Our data showed that PH patients required higher doses of vasopressors in the immediate post-intubation period, and increased vasopressor requirements were significantly associated with increased in-hospital mortality. While disease severity may have been a confounding factor, our findings suggest that maintaining hemodynamic stability after ETI in the PH population may be crucial. Tight blood pressure control and maintaining hemodynamic stability have often been underappreciated goals in airway securement.^25,26^ Systemic hypotension in PH patients should be avoided as reduced systemic vascular resistance cannot be compensated by increasing cardiac output when PVR is fixed.^5^ While intravenous fluid resuscitation may be a solution, fluids can worsen RV compromise and should be judiciously used.^20^ Thus, vasopressor use is crucial, and the expert consensus considers norepinephrine to be the first choice in pulmonary arterial hypertension,^5^ similar to what we observed in our cohort. While a few patients in our cohort were treated with phenylephrine, it is important to note that this medication can increase PVR,^27^ thus worsening hemodynamic compromise. Interestingly, while PH patients are known to be at risk of cardiac arrest due to hemodynamic collapse,^6,22^ these episodes were not seen in our cohort of patients despite higher vasopressor requirements. With our data, we believe future prospective studies on emergent intubation strategies in PH patients with acute right heart failure should focus on fluid and vasopressor management in the early post-intubation period.

It is well known that acute hypercapnia and elevated hydrogen ion concentration in the blood increase extracellular Ca^2+^ influx, thus vasoconstricting pulmonary circulation and subsequently worsening RV dysfunction.^28^ Within our subgroup analyses, increased post-intubation PaCO_2_ was significantly associated with in-hospital mortality in our PH patients: a finding not demonstrated in the control group. However, this may appear to be contradictory to the study by Hoeper *et al* who showed that baseline hypocapnia under PaCO_2_ 31.8 mmHg, a cutoff determined by receiver-operator curves, was significantly associated with increased mortality over 12 years.^29^ The authors argue that hyperventilation in PH patients may be due to decreased pulmonary perfusion (associated with decreased cardiac output), evidenced by an increased minute ventilation/carbon dioxide production (V’E/V’CO_2_ ratio). However, it is important to note that this study and others that explore baseline PaCO_2_ in pulmonary hypertension patients are conducted in chronic, stable outpatients instead of those who are acutely decompensating such as the patients in our study. Before intubation, our PH patients were more hypercapnic on average to 49.9 ± 25.1 mmHg, including PH patients who died (46.1 ± 19.1 mmHg). Regardless, our analyses did not demonstrate that pre-intubation hypercapnia was significantly associated with mortality; rather, an increase in post-intubation PaCO_2_ was observed in our PH patients who did not survive during their hospitalizations. Thus, we hypothesize that maintaining adequate ventilation and preventing increases in PaCO_2_ in the immediate post-intubation period may improve survival in the acute setting, though more robust statistical analyses are necessary.

Furthermore, current literature strongly suggests that severe hypercapnia with acidosis should be avoided in the absence of acute lung injury (ALI) and acute respiratory distress syndrome (ARDS), where permissive hypercapnia is the standard of care.^30^ However, preventing hypercapnia may not be as simple as increasing tidal volumes or respiratory rates, which could further impede RV outflow by gas trapping.^31^ The alternative strategy of lung recruitment maneuvers with positive end-expiratory pressure (PEEP) could increase lung volumes in pulmonary hypertension patients, thus worsening transpulmonary pressures and PVR.^30,32^ In a similar vein, low tidal volume ventilation should be utilized to prevent lung overdistension, which is employed in ARDS management.^33^ At 24 h post-intubation, the average PEEP in our study was slightly greater in the PH cohort, though this was not statistically significant (7.25 vs. 6.31, *P* = .15). These observations may suggest that PEEP should be carefully balanced alongside adequate ventilation to avoid potential increases in PVR and further RV dysfunction.^34^ Unfortunately, it was not possible to gather PVR data in this retrospective analysis; thus, future studies would benefit from also monitoring dynamic PVR changes relative to ventilatory adjustments.

Lastly, our cohort of PH patients had increased acute kidney injury in the early post-intubation period. PH patients are already at risk for impaired kidney function^35,36^ due to impaired baseline hemodynamics secondary to right heart failure.^37^ With the evidence that our PH cohort required increased vasopressor support as previously discussed, we hypothesize that AKI may be due to worsened hemodynamic instability in the immediate post-intubation period. This finding may further highlight the need to mitigate post-intubation hypotension. Additionally, PH patients may be more vulnerable to ventilator-induced kidney injury (VIKI). Although it is difficult to characterize the role of ventilatory settings in the pathogenesis of VIKI,^38,39^ we observed increased AKI in PH patients despite similar PEEP settings between our PH and control cohorts. It is well known that AKI is independently associated with increased mortality and would compound upon the mortality contributed by ETI and PH.^40,41^ Therefore, close attention to lung-protective ventilation and maintaining hemodynamic stability may be beneficial strategies to prevent AKI.^42^

Our study has several limitations. This is a retrospective analysis of nonconsecutive PH patients who demonstrated various indications for emergent ETI among our entire group of patients, which may carry implications in shaping short-term 24-h outcomes. Our PH patients were carefully selected to include PH Groups 1 and 3 with evidence of right heart failure and exclude patients with left cardiac disease. While this PH cohort is one of the larger groups studied thus far in the context of intubation, our sample size is limited within the context of a 15-year period of review. Our sample size is relatively limited because we had carefully selected our patients with well-documented pulmonary hypertension to devise a cohort that was as homogeneous as possible. Additionally, control patient matching using CCI did not lead to the most optimal matches as there are a few noteworthy discrepancies between cohorts such as indication for intubation as most PH patients were intubated for hypoxemia/hypoxia while control patients were intubated for mostly airway protection. Although propensity score weighting corrected most baseline patient characteristics, the statistical model may not perfectly balance all differences between cohorts. Though we have aimed for a 1:3 matched control cohort, we could only reach approximately a 1:2 match within our database. Our study did not take pulmonary vasodilators into account as they could not be compared to our control cohort; however, we recognize that pulmonary vasodilators are critical in acute PH management with their potential to reduce PVR.^43^ We used the classic definition of PH during our study: mPAP ≥ 25 mmHg;^4^ however, the classification was recently redefined to mPAP ≥ 20 mmHg.^44^ Our database did not provide all data points in short-term outcomes (24-h post-intubation) in patients who were analyzed before the year 2014 when our current electronic medical record system went live in our hospitals. This deficiency of specific data points affected 29 of our 48 PH patients (58.3%) and 1 of our 110 control patients (0.9%). Nevertheless, our primary mortality outcomes and ABG subgroup analyses were unaffected. Additionally, we did not have expiratory CO2 data to calculate dead space volume/tidal volume ratios which may contribute to our cohort's relative hypercapnia. In the future, we would like to explore how alveolar dead space may affect blood gases in the context of intubations. It is also important to note that the RSI protocol most likely improved over the past 16 years, suggesting improved outcomes for more recently intubated patients. While it was clear that the PH cohort had greater FiO_2_ requirements and lower P/F ratios, we did not have data on respiratory quotients, which would more accurately estimate alveolar O_2_.

Our study has a strength in that we exclusively selected patients requiring emergent ETI, focusing on a population with profound clinical deterioration during the study period. To date, we are unaware of another cohort study that directly compares short-term outcomes in PH patients requiring ETI with clinically matched, non-PH patients while also correcting for baseline differences. As we gain further insight into this vulnerable, immediate post-intubation period, these findings may be used to improve interventions and survival for PH patients who undergo intubation.

## Conclusion

Our findings demonstrated worsened outcomes in mechanically ventilated PH patients after emergent endotracheal intubation compared to similar patients without PH. More importantly, we showed that the first 24 h following intubation in PH patients represented a particularly vulnerable period where complications such as increased vasopressor requirements, hypercapnia, and AKI may affect long-term outcomes. With awareness of these early post-intubation complications, clinicians may and perhaps improve survival in PH patients who undergo emergent intubations.

## Supplemental Material

sj-docx-2-jic-10.1177_08850666221118839 - Supplemental material for Outcomes and Prognostic Factors of Pulmonary Hypertension Patients Undergoing Emergent Endotracheal Intubation Click here for additional data file. Supplemental material, sj-docx-2-jic-10.1177_08850666221118839 for Outcomes and Prognostic Factors of Pulmonary Hypertension Patients Undergoing Emergent Endotracheal Intubation by Andrew W. Hong, William Toppen, Joyce Lee, Holly Wilhalme, Rajan Saggar and Igor Z. Barjaktarevic in Journal of Intensive Care Medicine

sj-xlsx-3-jic-10.1177_08850666221118839 - Supplemental material for Outcomes and Prognostic Factors of Pulmonary Hypertension Patients Undergoing Emergent Endotracheal Intubation Click here for additional data file. Supplemental material, sj-xlsx-3-jic-10.1177_08850666221118839 for Outcomes and Prognostic Factors of Pulmonary Hypertension Patients Undergoing Emergent Endotracheal Intubation by Andrew W. Hong, William Toppen, Joyce Lee, Holly Wilhalme, Rajan Saggar and Igor Z. Barjaktarevic in Journal of Intensive Care Medicine

## List of Abbreviations

PH: pulmonary hypertension
RV: right ventricular
ETI: endotracheal intubation
CCI: Charlson Comorbidity Index
PVR: pulmonary vascular resistance
ICU: intensive care unit
mPAP: mean pulmonary artery pressure
ECMO: extracorporeal membrane oxygenation
RSI: rapid sequence intubation
BVM: bag-valve mask
NE: norepinephrine
ABG: arterial blood gas
GCS: Glasgow Coma Scale
HR: heart rate
RR: respiratory rate
MAP: mean arterial pressure
Hct: hematocrit
OR: odds ratio
IQR: interquartile range
ALI: acute lung injury
ARDS: acute respiratory distress syndrome
PEEP: positive end-expiratory pressure
AKI: acute kidney injury
VIKI: ventilator-induced kidney injury
NIPPV: noninvasive positive pressure ventilation
