# Supplementary material for: Outcomes and Prognostic Factors of Pulmonary Hypertension Patients Undergoing Emergent Endotracheal Intubation
Source: J Intensive Care Med. 2022 Aug 8;38(3):280–9. doi: 10.1177/08850666221118839 (PMC9806479; doi:10.1177/08850666221118839)
Supplement: sj-docx-2-jic-10.1177_08850666221118839 - Supplemental material for Outcomes and Prognostic Factors of Pulmonary Hypertension Patients Undergoing Emergent Endotracheal Intubation [file sj-docx-2-jic-10.1177_08850666221118839.docx]

**Supplemental Table S1. Odds of inpatient mortality with the number of simultaneous vasopressors with propensity score weighting.**

Effect of requiring more than one simultaneous vasopressor post-intubation (24 hours) on inpatient mortality with propensity score weighting. Separate univariate logistic regressions were run for each cohort: pulmonary hypertension, control, and total.

| Number of vasopresssors per group | OR | 95%CI | P |
| --- | --- | --- | --- |
| Pulmonary Hypertension |  |  |  |
| Number of Simultaneous Vasopressors |  |  |  |
| 1 vasopressor | 0.76 | 0.08-7.00 | 0.81 |
| ≥2 vasopressors | 12.0 | 1.40-104 | 0.023* |
|  |  |  |  |
| Control |  |  |  |
| Number of Simultaneous Vasopressors |  |  |  |
| 1 vasopressor | 2.65 | 0.92-7.68 | 0.07 |
| ≥2 vasopressors | 6.96 | 1.27-38.0 | 0.025* |
|  |  |  |  |
| Total Cohort |  |  |  |
| Number of Simultaneous Vasopressors |  |  |  |
| 1 vasopressor | 2.00 | 0.76-5.28 | 0.15 |
| ≥2 vasopressors | 9.33 | 2.72-31.9 | 0.000*** |

**Supplemental Table S2. Arterial blood gas values pre- and post-intubation between pulmonary hypertension and control cohorts.**

Documented arterial blood gas results immediately preceding their emergent intubation (pre-intubation) and immediately afterward (post-intubation). Pre-intubation values depicted are the latest blood gases preceding intubation, post-intubation values represent the earliest blood gases collected after intubation. All arterial gases were collected within 24 hours of the intubation event.

| Arterial Blood Gas Values | PH  (N=35) | Control  (N=87) | P |
| --- | --- | --- | --- |
| Pre-intubation ABGs |  |  |  |
| P_a_CO_2_, (mean ± SD), mmHg | 49.9 ± 25.1 | 41.1 ± 16.0 | 0.07 |
| P_a_O_2_ (mean ± SD), mmHg | 71.9 ± 36.9 | 108 ± 61.7 | 0.004 |
| pH, (mean ± SD) | 7.35 ± 0.12 | 7.40 ± 0.31 | 0.44 |
| Post-intubation ABGs |  |  |  |
| P_a_CO_2_, (mean ± SD), mmHg | 48.6 ± 18.7 | 40.6 ± 13.0 | 0.008 |
| P_a_O_2_ (mean ± SD), mmHg | 102 ± 72.7 | 167 ± 111 | 0.002 |
| pH, (mean ± SD) | 7.34 ± 0.13 | 7.35 ± 0.13 | 0.75 |
